# Assessment of Heart Rate Monitoring During Exercise With Smart Wristbands and a Heart Rhythm Patch: Validation and Comparison Study

**DOI:** 10.2196/52519

**Published:** 2023-12-14

**Authors:** Tse-Lun Wang, Hao-Yi Wu, Wei-Yun Wang, Chao-Wen Chen, Wu-Chien Chien, Chi-Ming Chu, Yi-Syuan Wu

**Affiliations:** 1 Division of Trauma and Surgical Critical Care Department of Surgery Kaohsiung Medical University Hospital, Kaohsiung Medical University Kaohsiung City Taiwan; 2 Graduate Institute of Medicine College of Medicine Kaohsiung Medical University Kaohsiung City Taiwan; 3 Department of Nursing Tri-Service General Hospital Taipei City Taiwan; 4 National Defense Medical Center and Department of Nursing School of Nursing Tri-Service General Hospital Taipei City Taiwan; 5 Department of Emergency Medicine Faculty of Medicine, College of Medicine Kaohsiung Medical University Kaohsiung City Taiwan; 6 Department of Medical Research Tri-Service General Hospital National Defense Medical Center Taipei City Taiwan; 7 School of Public Health National Defense Medical Center Taipei Taiwan; 8 Graduate Institute of Life Sciences National Defense Medical Center Taipei Taiwan; 9 Graduate Institute of Medical Sciences National Defense Medical Center Taipei Taiwan; 10 Big Data Research Center College of Medicine Fu-Jen Catholic University New Taipei City Taiwan; 11 Department of Public Health Kaohsiung Medical University Kaohsiung City Taiwan; 12 Department of Public Health China Medical University Taichung City Taiwan

**Keywords:** running, wearable device, photoplethysmography, heart rhythm patch, smart wristband

## Abstract

**Background:**

The integration of wearable devices into fitness routines, particularly in military settings, necessitates a rigorous assessment of their accuracy. This study evaluates the precision of heart rate measurements by locally manufactured wristbands, increasingly used in military academies, to inform future device selection for military training activities.

**Objective:**

This research aims to assess the reliability of heart rate monitoring in chest straps versus wearable wristbands.

**Methods:**

Data on heart rate and acceleration were collected using the Q-Band Q-69 smart wristband (Mobile Action Technology Inc) and compared against the Zephyr Bioharness standard measuring device. The Lin concordance correlation coefficient, Pearson product moment correlation coefficient, and intraclass correlation coefficient were used for reliability analysis.

**Results:**

Participants from a Northern Taiwanese medical school were enrolled (January 1-June 31, 2021). The Q-Band Q-69 demonstrated that the mean absolute percentage error (MAPE) of women was observed to be 13.35 (SD 13.47). Comparatively, men exhibited a lower MAPE of 8.54 (SD 10.49). The walking state MAPE was 7.79 for women and 10.65 for men. The wristband’s accuracy generally remained below 10% MAPE in other activities. Pearson product moment correlation coefficient analysis indicated gender-based performance differences, with overall coefficients of 0.625 for women and 0.808 for men, varying across walking, running, and cooldown phases.

**Conclusions:**

This study highlights significant gender and activity-dependent variations in the accuracy of the MobileAction Q-Band Q-69 smart wristband. Reduced accuracy was notably observed during running. Occasional extreme errors point to the necessity of caution in relying on such devices for exercise monitoring. The findings emphasize the limitations and potential inaccuracies of wearable technology, especially in high-intensity physical activities.

## Introduction

The current landscape of information technology, particularly in the realm of wearable technology, has seen remarkable advancements. These developments have facilitated continuous physiological monitoring, now made more accessible and user-friendly through compact sensing devices [1]. Modern smart wristbands exemplify this technological evolution. They are capable of tracking a variety of data, encompassing both movement (eg, speed and acceleration) and physiological information (eg, heart rate, resting heart rate, and body temperature), while also providing user feedback [2,3]. Notably, heart rate monitoring is achieved through the analysis of electrocardiography (ECG) and pulse waveform data. These readings are intrinsically linked to bioelectrophysiology and biomechanics, offering insightful reflections on an individual’s health status [4].

Currently, the majority of commercially available wearable devices leverage photoplethysmography (PPG) technology to gather physiological data. This method involves the use of light-emitting diodes to illuminate the skin, followed by the measurement of transmitted or reflected light via photodiodes. Such measurements are critical for assessing pulse pressure-induced changes in blood circulation [5]. Beyond heart rate, PPG technology has been expanded to monitor other physiological parameters like respiration and blood oxygen levels [6], and even individual blood glucose levels [1,7]. Numerous research initiatives, both within China and internationally, have extensively explored wearable smart mobile devices, resulting in significant findings [8,9]. These advancements present both opportunities and challenges in the ongoing development of smart wristbands.

The proliferation of wearable devices in fitness regimes is markedly evident, especially with the increasing popularity of sports wristbands during physical activities. However, there exists a notable variance in performance and accuracy across different brands. In military academies, where the use of such devices is on the rise, adherence to rigorous military standards is paramount. As a result, most of these devices are developed by local manufacturers. Validating their precision in heart rate measurement is essential and will also be beneficial for future considerations in selecting wearable devices. Consequently, we opted for a locally manufactured wristband, the Q-Band Q-69 developed by Mobile Action Technology Inc [10], for our validation and comparison purposes. This choice was guided by the intention to aid military units in selecting appropriate measurement devices for running-related activities in the future.

**Figure 1:**
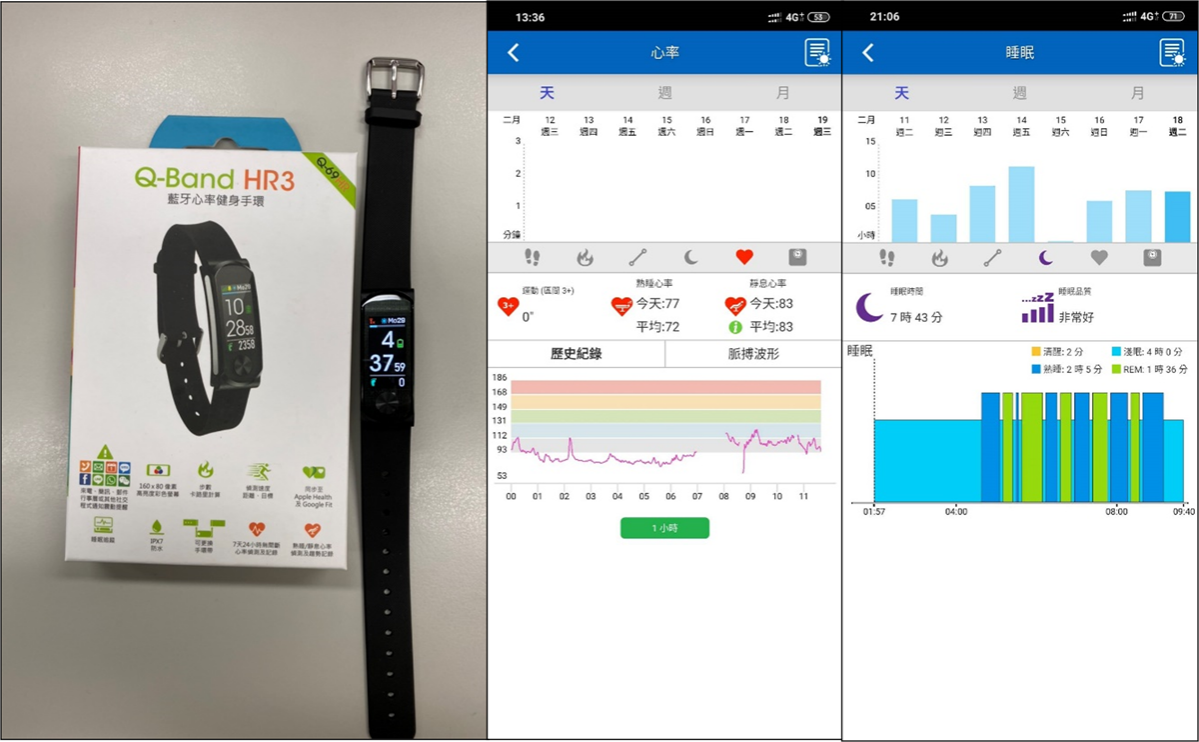
Q-Band HR3 (Q-69HR) device and graphical representation of heart rate and sleep records as displayed in the mobile app.

The Q-Band Q-69 wristband was used to gather data on heart rate and acceleration. It is adept at monitoring a variety of parameters, including movement patterns, heart rate, acceleration, daily exercise, and sleep patterns. For data synchronization and analysis, the i-gotU Life mobile app was used, which is available for users on both Google Play and the Apple App Store (Figure 1). The aim of this study is to compare the reliability of heart rate monitoring between chest straps and wearable wristbands.

## Methods

### Overview

Before the commencement of the 1600-meter running test, each participant’s preparation took place in the gym. This preparation included ensuring that 2 devices—the Zephyr BioPatch and the MobileAction Q-Band Q-69 wristband connected to an Android phone via Bluetooth (Figure 1)—were correctly fitted on each participant. The test began with the participants walking around a 400-meter track at a normal pace. Upon nearing the starting line, they activated the Zephyr and wristband by jumping, thereby initiating the 1600-meter running test.

In this study, a customized application was developed using the engineering firmware and Android application package for the Q-Band Q-69 smart wristband, provided by Mobile Action Technology Inc. The development process used Android Studio (Google), a versatile platform for app creation. This application was designed to extract heart rate and acceleration data recorded by the smart wristband. It enabled data exchange with mobile phones via Bluetooth pairing, facilitating the transmission of collected data to the research database. Additionally, the application supported the analysis of physiological data generated during exercise, incorporating data upload function for long-term data tracking. The acceleration data from both the wristband and the Zephyr BioHarness 3.0 were collected using the tri-axial accelerometers embedded within these devices.

**Figure 2:**
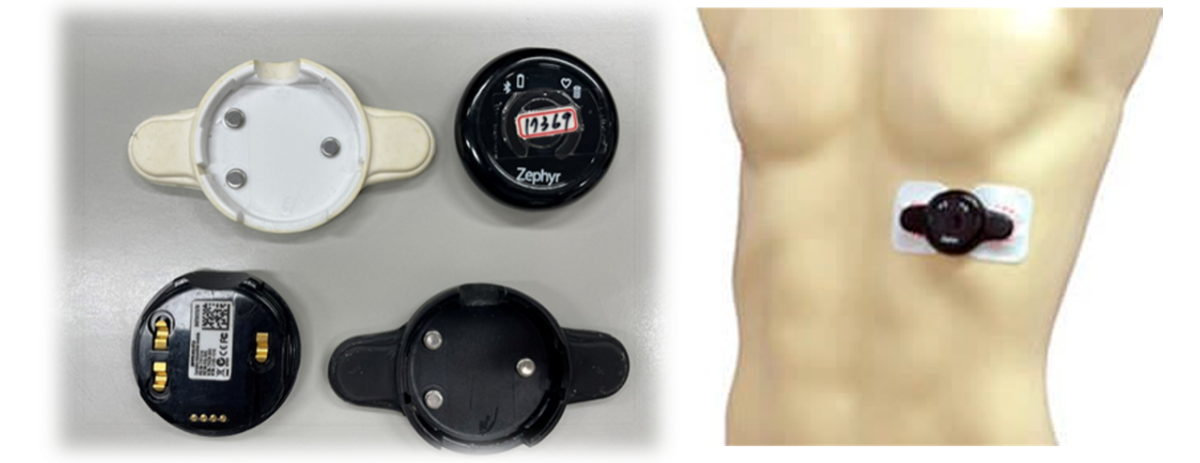
Schematic illustration of the BioHarness 3.0 BioPatch device and its placement.

For the purpose of collecting physiological parameters (heart rate, acceleration, and activity levels), the Medtronic Zephyr BioHarness 3.0 BioPatch was used. The use of this BioPatch necessitates its integration with ECG patches. In this study, Ambu BlueSensor T (Ambu A/S) ECG patches, specifically designed for exercise monitoring (Figure 2), were used. A total of 2 of these ECG patches were connected to each end of the BioPatch via clip-on buttons and then affixed to the skin near the heart area. These patches recorded changes in physiological parameters, with the collected data being logged by the BioPatch device.

### Statistical Analyses

In this study, Microsoft Office 365 Excel was used for data preprocessing. SPSS 26 software (IBM Corp) and MedCalc (MedCalc Software) statistical software were used for variable input and data analysis. The significance level of this study was set at α=.05.

To compare the accuracy and reliability between the standard measuring device (Zephyr Bioharness) and the smart wristband (Q-Band Q-69, the test device), Lin concordance correlation coefficient (CCC), Pearson product moment correlation coefficient (PPMCC), and intraclass correlation coefficient (ICC) (using a single-measure, 2-way mixed mode and absolute consistency) were used to interpret the reliability test results. Previous research findings indicated that a CCC value greater than 0.80 indicates acceptable reliability [11]. Leung et al [12] proposed that an ICC value between 0.75 and 0.90 indicates high reliability [12] and that an ICC value between 0.50 and 0.75 indicates medium reliability [13].

### Paired Difference Analysis

Mean absolute error (MAE) and mean absolute percentage error (MAPE) were used to assess the differences between the standard measurements and the values measured by the test device at different exercise stages. MAPE is calculated as follows: (heart rate data per second for the Zephyr Bioharness – heart rate data per second for the Q-Band Q-69 smart wristband) / heart rate data per second for the Zephyr Bioharness. Previous research has shown that an error of less than 10% indicates that the result is reliable [13,14].

### Bland-Altman Plot Analysis

This study used Bland-Altman plot analysis to assess the agreement between measurement values for the standard measurement device (Zephyr Bioharness) and the PPG-based smart wristband (Q-Band Q-69) and analyze the mean difference and the 95% limits of agreement (LoA). Previous research results suggest that data points within the 95% LoA should be distributed normally [15].

### Ethical Considerations

Ethics approval for this research was granted by the Trial Committee of the Tri-Service General Hospital in Taiwan (C202005175). All data in this study were handled with strict confidentiality. The collection, analysis, and storage processes were conducted anonymously to ensure participant privacy and data integrity. Informed consent was obtained from all individuals involved in the study.

## Results

Volunteer participants for this study were recruited from a medical school in Northern Taiwan between January 1, 2021, and June 31, 2021. All participants attended an initial briefing session where they were informed about the study’s objectives and procedures.

This study evaluated the accuracy and reliability of the MobileAction Q-Band Q-69 smart wristband by comparing it with a standard measurement device, the Zephyr BioHarness. Out of the 154 initial participants, 20 participants (10 women and 10 men) were selected based on stringent criteria. These included ensuring that both the heart rate quality and system quality of the Zephyr BioHarness exceeded 95% and that the wristband’s heart rate readings displayed no irregularities. The Zephyr BioHarness’s data incorporates advanced algorithms for assessing heart rate and system quality [16]. The majority of participants were excluded due to unreliable device data, which manifested as missing, or excessively high or low heart rate readings when compared to the Zephyr BioHarness. Consequently, only 20 cases met the criteria for inclusion. According to the formula proposed by Zou [17], a minimum sample size of 8 participants is required to attain a target efficacy of 0.90.

The evaluation of heart rate data accuracy for the MobileAction Q-Band Q-69 smart wristband in Tables 1 and 2 encompasses MAE, MAPE, and correlation analysis across various activity stages. The results indicate gender-specific variations in MAPE values during different activities. During the running phase, the MAPE for women was recorded at 13.35 (SD 13.47). In contrast, for men, the MAPE was lower at 8.54 (SD 10.49). In the walking state, the MAPE for women was 7.79 (SD 8.59), while for men, it was higher at 10.65 (SD 16.55). Notably, in all other activity states assessed, the wristband’s MAPE remained consistently below 10%, indicating a generally high level of accuracy in these conditions.

The study’s findings on the MobileAction Q-Band Q-69 revealed distinct patterns in CCCs and PPMCCs between genders. For women, the CCCs were observed as follows: an overall coefficient of 0.564 (Table 2), with specific activities yielding coefficients of 0.564 for walking, 0.058 for running, and 0.651 during the cooldown phase. In contrast, men exhibited higher CCCs, with an overall coefficient of 0.796, and activity-specific coefficients of 0.197 for walking, 0.481 for running, and 0.896 for the cooldown.

Additionally, the study assessed the PPMCCs, revealing a general coefficient of 0.625 for women, encompassing 0.595 for walking, 0.106 for running, and 0.656 for the cooldown phase. The men demonstrated a higher overall PPMCC of 0.808, with walking, running, and cooldown coefficients of 0.206, 0.588, and 0.937, respectively. These findings underscore significant gender-based variations in the performance metrics of the MobileAction Q-Band Q-69.

**Figure 3:**
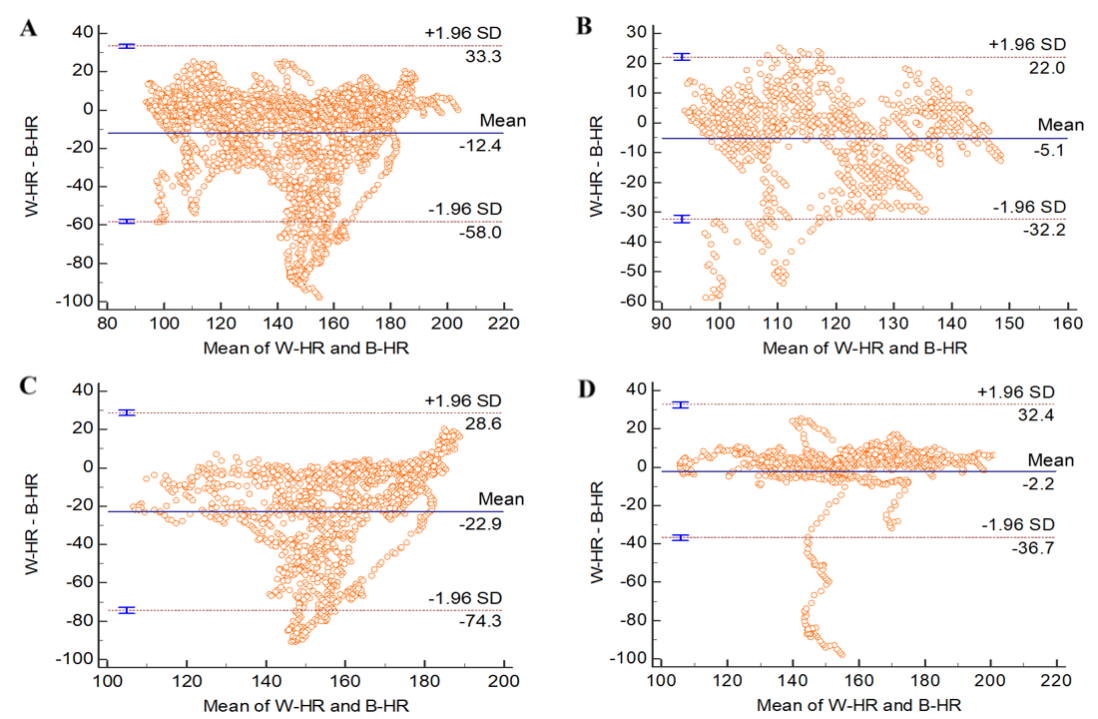
Bland-Altman plots for the heart rate data of women at different stages. B-HR: Zephyr BioHarness heart rhythm; W-HR: Q-Band Q-69 smart wristband heart rhythm.

**Figure 4:**
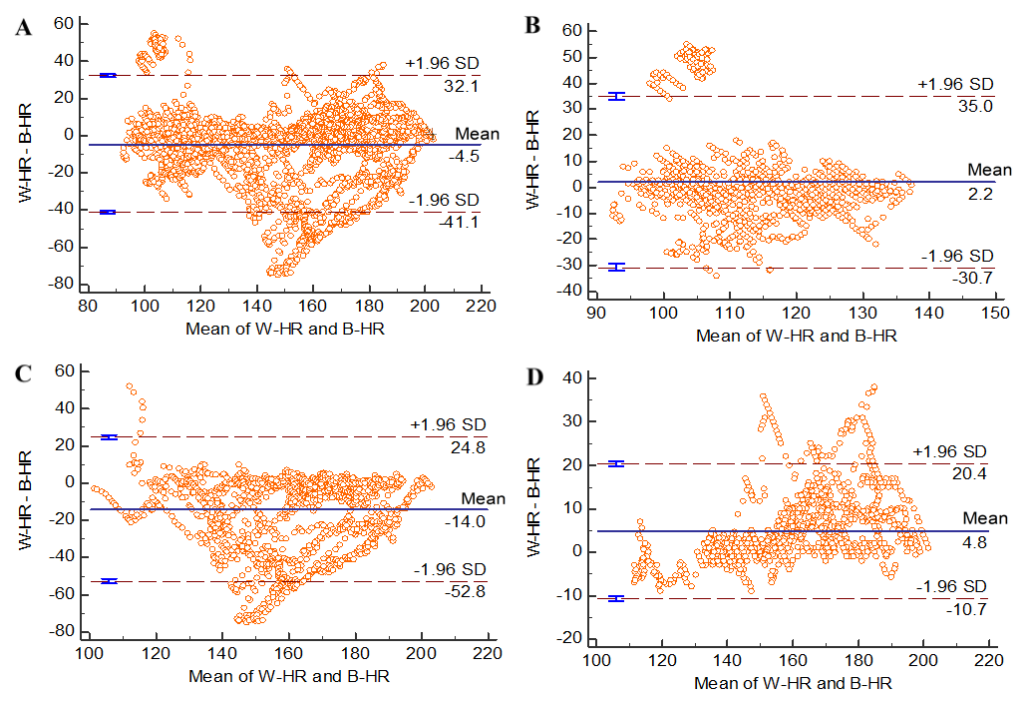
Bland-Altman plots for the heart rate data of men at different stages. B-HR: Zephyr BioHarness heart rhythm; W-HR: Q-Band Q-69 smart wristband heart rhythm.

The Bland-Altman plot analysis, illustrated in Figures 3 and 4, elucidates the mean differences in heart rate measurements between the MobileAction Q-Band Q-69 smart wristband and the Zephyr BioHarness device. This analysis also encompasses the level of agreement within a 95% LoA for both men and women.

Detailed Bland-Altman analysis data are presented in Table 1. For women, the overall (Figure 3A) mean difference was –12.4 (95% LoA –58.0 to 33.3). During walking (Figure 3B), the mean difference was –5.1 (95% LoA –32.2 to 22.0). In the running phase (Figure 3C), a more pronounced mean difference of –22.9 (95% LoA –74.3 to 28.6) was observed. Finally, during the cooldown (Figure 3D), the mean difference was –2.2 (95% LoA –36.7 to 32.4). Notably, in the walking stage (Figure 3B), a small cluster of data for women exceeded SD 1.96 in the lower left corner of the plot. Furthermore, during cool down (Figure 3D), a distinct group of data fell below SD 1.96.

For men, the overall (Figure 4A) mean difference was –4.5 (95% LoA –41.1 to 32.1). In the walking stage (Figure 4B), the mean difference was slightly positive at 2.2 (95% LoA –30.7 to 35.0). During running (Figure 4C), the mean difference was –14.0 (95% LoA –52.8 to 24.8), and in the cooldown phase (Figure 4D), it was 4.8 (95% LoA–10.7 to 20.4). It was observed that in labels B and C among men, there were fewer data points exceeding SD 1.96.

**Table 1:** Mean absolute percentage error (MAPE) and Bland-Altman analysis of heart rate readings of women and men in different activity phases.

State		Seconds	Device	MAPE analysis, mean (SD)		Bland-Altman analysis
				MAE^a^ (bpm)	MAPE	Mean difference (95% LoA^b^)
**Women**						
	Walking	1800	Q-Band	9.63 (11.15)	7.79 (8.59)	–5.1 (–32.2 to 22.0)
	Running	3000	Q-Band	23.86 (25.33)	13.35 (13.47)	–22.9 (–74.3 to 28.6)
	Cooldown	1800	Q-Band	7.61 (16.05)	4.57 (8.63)	–2.2 (–36.7 to 32.4)
	Overall	6600	Q-Band	15.55 (21.31)	9.44 (11.70)	–12.4 (–58.0 to 33.3)
**Men**						
	Walking	1800	Q-Band	10.30 (13.41)	10.65 (16.55)	2.2 (–30.7 to 35.0)
	Running	3000	Q-Band	15.11 (18.97)	8.54 (10.49)	–14.0 (–52.8 to 24.8)
	Cooldown	1800	Q-Band	6.04 (7.07)	3.83 (4.51)	4.8 (–10.7 to 20.4)
	Overall	6600	Q-Band	11.33 (15.51)	7.83 (11.70)	–4.5 (–41.1 to 32.1)

^a^MAE: mean absolute error.

^b^LoA: limits of agreement.

**Table 2:** Correlation analysis of heart rate data in different activity stages.

State		Seconds	Device	CCC^a^	ICC^b^	PPMCC^c^
**Women**						
	Walking	3600	Q-Band	0.564	0.565	0.595
	Running	6000	Q-Band	0.058	0.058	0.106
	Cooldown	3600	Q-Band	0.651	0.651	0.656
	Overall	13,200	Q-Band	0.564	0.564	0.625
**Men**						
	Walking	3600	Q-Band	0.197	0.197	0.206
	Running	6000	Q-Band	0.481	0.482	0.588
	Cooldown	3600	Q-Band	0.896	0.896	0.937
	Overall	13,200	Q-Band	0.796	0.796	0.808

^a^CCC: Lin concordance correlation coefficient.

^b^ICC: intraclass correlation coefficient.

^c^PPMCC: Pearson product moment correlation coefficient.

## Discussion

### Principal Findings

In this study, the Q-Band Q-69 smart wristband provided by Mobile Action Technology Inc, which has been approved by the National Communications Commission of the Republic of China, was used [18]. At present, there is no relevant academic report on the accuracy or reliability of the heart rate detection results obtained through the Q-Band Q-69 smart wristband. Therefore, this is the first study to assess the accuracy and reliability of the Q-Band Q-69 smart wristband in measuring heart rate. Small wearable devices currently on the market (eg, Apple Watch, Garmin, Samsung, Fitbit, and Mi Band by Xiaomi) usually provide accurate heart rate data [13,19-24].

Diverse statistical analysis methodologies, such as MAE, MAPE, CCC, ICC, PPMCC, and Bland-Altman plot analysis, have been used in previous studies to assess correlation [13,14,21,23]. This study’s use of per-second heart rate recording, mirroring the approach of the Zephyr BioHarness device, marks a significant enhancement over prior research that typically sampled heart rate data at 15-second or minute intervals [25,26]. This more granular data collection enables better alignment of time intervals between 2 different devices, thus improving the accuracy of comparative analyses.

The MAPE values for the Q-Band Q-69 smart wristband in our study were 8.54% (n=10.49) for men while running, 13.35% (n=13.47) for women in the running state, 10.65% (n=16.55) for men in the walking state, and below 10% for other states, suggesting acceptable accuracy and reliability. This finding contrasts with a study on the Apple Watch 1, which reported a MAPE range of 6.33% to 10.69% during stationary bike exercises [20], attributed to minimal upper body movement. Our study observed higher MAPE values for running (13.35 for women and 8.54 for men), likely due to increased motion affecting the wrist-worn device. These observations underscore the importance of including a variety of physical activities in future research to understand device performance across different exercise conditions.

Comparatively, other studies reported MAPE values of 5.86% for the Apple Watch 3 and 5.96% for the Fitbit Charge 2 in 24-hour heart rate monitoring [22]. Additionally, the Garmin wristband showed a MAPE of 3.77% for people who are young and 4.73% for people who are older, while the Mi Band recorded 7.69% and 6.04%, respectively, for these age groups [13]. A recent study highlighted the Fitbit Charge 4’s superior MAPE values during rest and sedentary activities, and the Galaxy Watch Active2’s better performance during transitions from low- to high-intensity activities [23].

Research literature indicates that a CCC above 0.80 is generally indicative of acceptable reliability [11]. Leung et al [12] have established that an ICC ranging between 0.75 and 0.90 signifies high reliability, while an ICC from 0.50 to 0.75 suggests medium reliability [13]. In this study, the overall CCC for the MobileAction Q-Band Q-69 smart wristband was 0.564 for women and 0.796 for men. These results imply that the wristband exhibits moderate reliability in heart rate monitoring for men, but less so for women. Notably, in the running state for women, both the CCC and ICC were as low as 0.058, indicating poor reliability in this specific activity.

This observation of reduced reliability in certain activity states is corroborated by findings from other wearable devices. For example, the Fitbit Charge 2 displayed an ICC of only 0.21 during cycling activities [27]. This lower ICC might be attributable to variations in reliability across different physical activities. Supporting this, Shcherbina et al [28] observed that the Fitbit Surge demonstrated greater accuracy during cycling compared to walking or running. Additionally, the same study highlighted that the CCC and ICC for the Fitbit Surge in men during the cooldown phase postrunning reached 0.896, representing the most optimal correlation results among all the analyzed activities. This finding aligns with reports by Nissen et al [23] concerning the Fitbit Charge 4 and the Galaxy Watch Active2, underscoring the importance of considering activity-specific performance in wearable heart rate monitoring devices.

In the Bland-Altman analysis conducted in this study, the mean differences in heart rate measurements were discerned for both genders. Specifically, for men, the overall mean difference was 4.5 (95% LoA –41.1 to 32.1), whereas for women it was 12.4 (95% LoA 58.0 to 33.3). These findings suggest that the MobileAction Q-Band Q-69 smart wristband tends to underestimate heart rates, a trend consistent with previous research findings [13,29,30]. Notably, the wristband exhibited significant variance in heart rate measurements during transitions between different activities, with a reduced discrepancy observed when participants remained stationary.

Past studies have highlighted the potential influence of sex on PPG waveform results [31,32], though there are ongoing debates and differing perspectives regarding sex differences in PPG outcomes [33,34]. Given these unresolved discussions, it is advisable for users to exercise caution when monitoring heart rate data during physical activities using PPG-based smart wristbands [13]. This caution is especially pertinent in light of the observed underestimation of heart rates by such devices, as revealed in our analysis.

### Research Limitations

This study introduces a notable advantage by incorporating the usage of a novel wearable device, the Zephyr BioPatch, alongside the MobileAction Q-Band Q-69 smart wristband. The use of the Q-Band Q-69 is particularly beneficial due to the ease of measurements it offers, enhanced by its diverse measurement capabilities facilitated through advanced application development. However, a limitation of this study is its exclusive comparison of the smart wristband with a Taiwan-manufactured device. Future research should expand this scope by comparing the Q-Band Q-69 with various other types of wristbands. Such comparative analyses are essential to comprehensively understand the performance differences across a wider range of wearable devices. This approach will not only validate the findings of this study but also contribute to a broader understanding of wearable technology’s capabilities in different contexts.

### Conclusions

The findings of this study reveal a notable variation in the measurement accuracy of the MobileAction Q-Band Q-69 smart wristband, particularly influenced by gender and the nature of the physical activity. A significant decrease in accuracy was observed during running activities. Additionally, there were instances where the wristband produced extreme errors under certain, as yet unidentified, conditions. These observations underscore the need for users to exercise caution regarding the reliability of exercise monitoring performance when using smart wristbands. This study’s results highlight the importance of understanding the limitations and potential inaccuracies inherent in wearable technology, especially in scenarios involving vigorous physical activities.

## Abbreviations

CCC: concordance correlation coefficient
ECG: electrocardiography
ICC: intraclass correlation coefficient
LoA: limits of agreement
MAE: Mean absolute error
MAPE: mean absolute percentage error
PPG: photoplethysmography
PPMCC: Pearson product moment correlation coefficient
